# Paradoxical pulmonary hemorrhage associated with hemocoagulase batroxobin in a patient with hemoptysis: A CARE-compliant case report

**DOI:** 10.1097/MD.0000000000024040

**Published:** 2021-01-29

**Authors:** Tae-Ok Kim, Min-Suk Kim, Bo Gun Kho, Ha Young Park, Yong-Soo Kwon, Yu-Il Kim, Sung-Chul Lim, Hong-Joon Shin

**Affiliations:** Department of Internal Medicine, Chonnam National University Hospital, Gwangju, South Korea.

**Keywords:** hemocoagulase, hypofibrinogenemia, pulmonary hemorrhage

## Abstract

**Rational:**

Hemocoagulase, a hemostatic, is used in patients with trauma, gastrointestinal bleeding, or pulmonary hemorrhage or those undergoing surgery. However, paradoxical bleeding after hemocoagulase administration is not considered a clinically significant adverse effect. Here, we report a case of paradoxical pulmonary hemorrhage associated with hypofibrinogenemia after administration of the hemocoagulase batroxobin in a patient with hemoptysis.

**Patient concerns:**

An 86-year-old woman complained of hemoptysis during hospitalization with organophosphate poisoning. Hemocoagulase was administered to manage bleeding; however, bleeding signs, such as hemoptysis, massive epistaxis, and ecchymosis, recurred.

**Diagnoses:**

The patient was diagnosed with acquired hypofibrinogenemia on the basis of the reduced plasma fibrinogen level after hemocoagulase administration and lack of other causes of bleeding.

**Intervention:**

Hemocoagulase administration was discontinued, and fibrinogen-containing plasma products were administered.

**Outcomes:**

The plasma fibrinogen level normalized and bleeding signs did not recur.

**Lessons:**

It is necessary to measure plasma fibrinogen levels regularly in patients undergoing hemocoagulase administration and discontinue its administration when acquired hypofibrinogenemia is detected.

## 1 Introduction

Hemocoagulase, a thrombin-like enzyme, forms fibrin clots, producing a hemostatic effect.^[1]^ It is used to reduce bleeding and transfusion requirements in patients undergoing surgery or those with trauma.^[2–4]^ Although its effect is unclear, it is also used in patients with gastrointestinal bleeding or pulmonary hemorrhage.^[5–7]^ However, paradoxical bleeding after hemocoagulase administration is not considered a clinically significant adverse effect. Here, we report a case of paradoxical pulmonary hemorrhage associated with hypofibrinogenemia after administration of the hemocoagulase batroxobin in a patient with hemoptysis.

## 2 Methods

This study was approved by the Institutional Review Board of the Chonnam National University Hospital (the number of approval: CNUH-EXP-2020-206). Written informed consent was obtained from the patient for publication of this case report and any accompanying images.

## 3 Case presentation

An 86-year-old woman presented to the emergency room (ER) with irritability. She had a medical history of hypertension and dementia. She had drunk approximately 100 ml of an insecticide 4 hours before presentation to the ER and undergone gastric lavage with a fluid volume of approximately 5 L at a local clinic. The insecticide contained chlorpyrifos, an organophosphate.

On arrival at the ER, her blood pressure was 160/100 mm Hg, body temperature was 36.9°C, heart rate was 110 beats/minutes, and respiratory rate was 20 breaths/minutes. Chest examination revealed no crackles or wheezing sounds. Initial plain chest radiography showed non-specific findings. Electrocardiography revealed sinus tachycardia at a heart rate of 117 beats/minutes.

Her white blood cell count was high at 19.4 × 10^3^/mm^3^ (reference range, 4.8–10.8 × 10^3^/mm^3^) and neutrophil percentage was 90.9%. Her hemoglobin level was 13.6 g/dl and platelet count was 416 × 10^3^/mm^3^. Her C-reactive protein level, electrolyte levels, renal and liver function study results, and coagulation profile were within normal limits. She was diagnosed with organophosphate poisoning on the basis of the plasma red blood cell (RBC) acetylcholinesterase activity of 4,763 U/L (reference range, 11,188–16,698 U/L).

The patient was administered pralidoxime, an antidote to organophosphate poisoning, for 5 days. Her plasma RBC acetylcholinesterase activity had recovered to 11,529 U/L on day 6. However, she was placed on mechanical ventilation and hemodialysis because of acute hypercapnic respiratory failure and acute kidney injury on day 5. After intensive care, extubation was performed on day 19, and hemodialysis was stopped on day 32.

**Figure 1:**
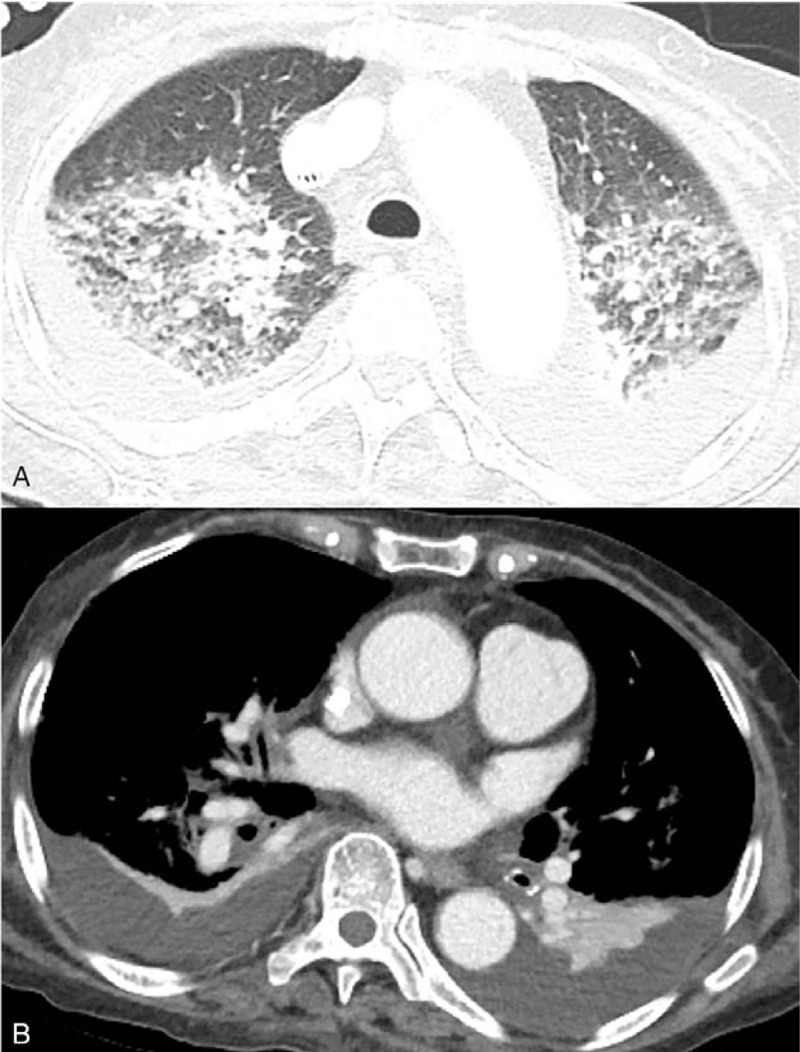
Chest computed tomography reveals patchy ground-glass opacities and consolidation with interlobular septal thickening in the dependent portion of the bilateral lungs (A). Moderate pleural effusion is noted in the bilateral lungs (B).

**Figure 2:**
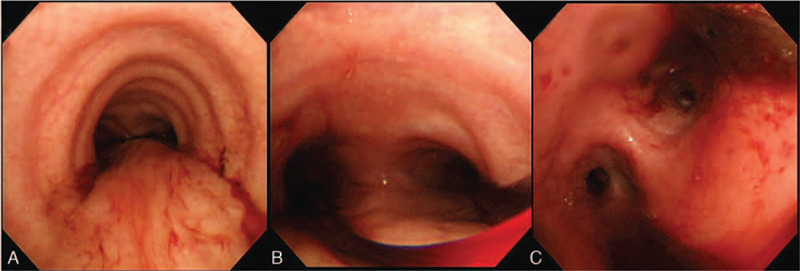
Fiberoptic bronchoscopy reveals diffuse pulmonary hemorrhage at the bilateral bronchial trees. (A) Mid-tracheal level; (B) Cranial level; (C) Distal left main bronchial level.

**Figure 3:**
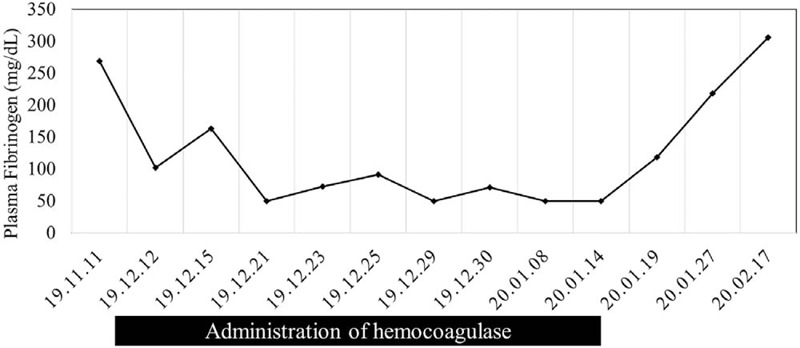
Plasma fibrinogen level throughout the patients hospitalization period. It had decreased during hemocoagulase administration and recovered after stopping hemocoagulase administration. (Date for hemocoagulase start was Dec 11, 2019. Date for hemocoagulase discontinuation was Jan 14, 2020.).

The patient complained of mild hemoptysis on day 41. Chest computed tomography showed pulmonary edema and bilateral pleural effusions (Fig. 1). The hemocoagulase batroxobin and furosemide were administered intravenously. Her platelet count was 129 × 10^3^/mm^3^ and prothrombin and activated partial thromboplastin times were within normal limits on day 42. However, the plasma fibrinogen level had decreased to 102 mg/dl. She received 8 U of cryoprecipitates. However, hemoptysis recurred on days 46 and 52. Although follow-up chest computed tomography showed improved pulmonary edema and pleural effusions, bronchoscopy revealed moderate pulmonary hemorrhage in bilateral bronchial trees (Fig. 2). Intravenous hemocoagulase was administered continuously to control pulmonary hemorrhage. She also received fresh frozen plasma (FFP) and cryoprecipitates because of hypofibrinogenemia. Her hemoptysis improved, but massive epistaxis and ecchymosis on her bilateral buttocks were developed on days 60 and 70, respectively. Hypofibrinogenemia was observed persistently without evidence of disseminated intravascular coagulation (DIC) or hemodilution (Fig. 3). After a literature review revealed the association of hemocoagulase administration with acquired hypofibrinogenemia, hemocoagulase administration was discontinued. The plasma fibrinogen level recovered dramatically, and no further bleeding signs were observed.

## 4 Discussion

Here, we reported a case of paradoxical bleeding after the hemocoagulase batroxobin was administered to manage pulmonary hemorrhage. Hemocoagulase, a thrombin-like enzyme, releases fibrinopeptide A, which forms fibrin clots.^[1]^ It reduces the bleeding time and transfusion requirements in patients undergoing surgery or with trauma.^[2,4]^ In addition, hemocoagulase administration in critical neonates reduces pulmonary hemorrhage and mortality.^[8,9]^ In patients with pulmonary hemorrhage, hemocoagulase can be used as a systemic hemostatic drug.^[5,7]^

Acquired hypofibrinogenemia is a condition of decreased fibrin number in blood associated with secondary causes. It is most frequently caused by hemodilution (massive transfusion) and consumption of clotting factors (sepsis with DIC).^[10]^ Advanced liver disease is also associated with acquired hypofibrinogenemia, which reduces the synthesis of fibrinogen in the liver.^[10]^ Because hemocoagulase releases fibrinogen to form fibrin clots, it can deplete fibrinogen levels. In patients undergoing polypectomy, fibrinogen levels decrease after hemocoagulase administration.^[11]^ In 11 patients with hematologic diseases, comparison of the plasma fibrinogen level before and after hemocoagulase administration showed a statistically significant decrease.^[12]^ In our case, the plasma fibrinogen level had decreased after hemocoagulase administration. In particular, the plasma fibrinogen level was <50 mg/dl when bleeding signs were observed.

Traditionally, in patients with hypofibrinogenemia, transfusions were performed when the plasma fibrinogen level was <100 mg/dl.^[13]^ However, recently, the threshold has been extended to 150 or 200 mg/dl.^[14]^ FFP and cryoprecipitates are usually used to replace fibrinogen in patients with hypofibrinogenemia. However, in the present case, the plasma fibrinogen level did not recover despite continuous FFP and cryoprecipitate replacements. However, after discontinuing hemocoagulase administration, the plasma fibrinogen level recovered without additional transfusions. As in our case, in a previous study, improvement of clinical symptoms was noted after discontinuing hemocoagulase administration in patients with hypofibrinogenemia and gastrointestinal bleeding who did not improve with replacement of fibrinogen-containing plasma products with hemocoagulase administration.^[6]^ Therefore, it is more important to discontinue hemocoagulase administration than to transfuse fibrinogen-containing plasma products in patients with hypofibrinogenemia and bleeding.

Predisposing factors for acquired hypofibrinogenemia after hemocoagulase administration have not been identified. A study has shown that hemocoagulase administration is a risk factor for fibrinogen deficiency in patients who underwent resection of an intracranial tumor.^[15]^ In that study, the total dose of administered hemocoagulase was significantly higher in the fibrinogen deficiency group than in the non-fibrinogen deficiency group.^[15]^ Therefore, higher doses of hemocoagulase may be a risk factor for the development of acquired hypofibrinogenemia.

## 5 Conclusions

Administration of hemocoagulase is associated with acquired hypofibrinogenemia, which may develop or promote bleeding. It is necessary to measure plasma fibrinogen levels regularly in patients undergoing hemocoagulase administration and discontinue its administration when acquired hypofibrinogenemia is detected.

## Author contributions

**Conceptualization:** Hong-Joon Shin.

**Data curation:** Tae-Ok Kim, Bo Gun Kho, Min-Seok Kim, Ha Young Park, Hong-Joon Shin.

**Formal analysis:** Tae-Ok Kim, Hong-Joon Shin.

**Funding acquisition:** Hong-Joon Shin.

**Resources:** Tae-Ok Kim, Bo Gun Kho, Min-Seok Kim, Ha Young Park.

**Supervision:** Yong-Soo Kwon, Yu-Il Kim, Sung-Chul Lim.

**Validation:** Yong-Soo Kwon, Yu-Il Kim, Sung-Chul Lim.

**Writing – original draft:** Tae-Ok Kim.

**Writing – review & editing:** Tae-Ok Kim, Hong-Joon Shin.

## Abbreviations

Abbreviations: DIC = disseminated intravascular coagulation, ER = emergency room, FFP = fresh frozen plasma, RBC = red blood cell.

## References

[1] Vu TT Stafford AR Leslie BA. Batroxobin binds fibrin with higher affinity and promotes clot expansion to a greater extent than thrombin. J Biol Chem 2013;288:16862–71. 23612970 10.1074/jbc.M113.464750 PMC3675619

[2] Qiu M Zhang X Cai H. The impact of hemocoagulase for improvement of coagulation and reduction of bleeding in fracture-related hip hemiarthroplasty geriatric patients: a prospective, single-blinded, randomized, controlled study. Injury 2017;48:914–9. 28238301 10.1016/j.injury.2016.11.028

[3] Nagabhushan RM Shetty AP Dumpa SR. Effectiveness and safety of batroxobin, tranexamic acid and a combination in reduction of blood loss in lumbar spinal fusion surgery. Spine (Phila Pa 1976) 2018;43:E267–73. 28678111 10.1097/BRS.0000000000002315

[4] Yao YT Yuan X Fang NX. Hemocoagulase reduces postoperative bleeding and blood transfusion in cardiac surgical patients: a PRISMA-compliant systematic review and meta-analysis. Medicine (Baltimore) 2019;98:e18534. 31876750 10.1097/MD.0000000000018534 PMC6946274

[5] Lee BR Yu JY Ban HJ. Analysis of patients with hemoptysis in a tertiary referral hospital. Tuberc Respir Dis (Seoul) 2012;73:107–14. 23166543 10.4046/trd.2012.73.2.107 PMC3492374

[6] Zhang H. The effects of hemocoagulase on coagulation factors in an elderly patient with upper gastrointestinal hemorrhage: a case report. Curr Drug Saf 2019;14:230–2. 31124425 10.2174/1574886314666190524093711 PMC6865287

[7] Jin F Li Q Bai C. Chinese expert recommendation for diagnosis and treatment of massive hemoptysis. Respiration 2020;99:83–92. 31509823 10.1159/000502156

[8] Shi Y Zhao J Tang S. Effect of hemocoagulase for prevention of pulmonary hemorrhage in critical newborns on mechanical ventilation: a randomized controlled trial. Indian Pediatr 2008;45:199–202. 18367764

[9] Lodha A Kamaluddeen M Akierman A. Role of hemocoagulase in pulmonary hemorrhage in preterm infants: a systematic review. Indian J Pediatr 2011;78:838–44. 21210254 10.1007/s12098-010-0326-4

[10] Besser MW MacDonald SG. Acquired hypofibrinogenemia: current perspectives. J Blood Med 2016;7:217–25. 27713652 10.2147/JBM.S90693 PMC5045218

[11] Zhou HB. Hypofibrinogenemia caused by hemocoagulase after colon polyps excision. Am J Case Rep 2017;18:291–3. 28325889 10.12659/AJCR.902059 PMC5373817

[12] Linglong X Dijiong W. Prolonged hemocoagulase agkistrodon halys pallas administration induces hypofibrinogenemia in patients with hematological disorders: a clinical analysis of 11 patients. Indian J Hematol Blood Transfus 2018;34:322–7. 29622877 10.1007/s12288-017-0859-z PMC5884981

[13] Levy JH Welsby I Goodnough LT. Fibrinogen as a therapeutic target for bleeding: a review of critical levels and replacement therapy. Transfusion 2014;54:1389–405. quiz 1388. 24117955 10.1111/trf.12431

[14] Levy JH Goodnough LT. How I use fibrinogen replacement therapy in acquired bleeding. Blood 2015;125:1387–93. 25519751 10.1182/blood-2014-08-552000

[15] Wei N Jia Y Wang X. Risk factors for postoperative fibrinogen deficiency after surgical removal of intracranial tumors. PLoS One 2015;10:e0144551. 26658430 10.1371/journal.pone.0144551 PMC4676605

